# 

**Published:** 1894-04-21

**Authors:** 


CONTENTS.

**School Fevers** - - - - - - - - - - - - 45

**Around the Hospitals** - - - - - - - - - 46

**On Modern Progress in Ophthalmic Medicine and Surgery.** By Robert Brudenell Carter - - - 47

**Studies in Applied Sociology.** VII.—The Homes of England 48

**The Psychologist in History.** I.—Madness or What? - - 49

**The First Century of English Medical Literature.** II. 50

**Annotations.**—A New Dermatological Society—Diphtheria at Leavesden—Huxley on Head and Heart - - - - 51

**Notes and News** - - - - - - - - - - 52

**Scraps and Gleanings** - - - - - - - - 64

**The Book World of Medicine and Science** - - - - 65

**Medical Vacancies and Appointments** - - - - - 66

**Medical Progress and Hospital Clinics—**
How to Avoid the Troubles which are due to Uric Acid. II. - 53
Treatment of Club-Foot - - - - - - - - - 54
Tinea Tonsurans - - - - - - - - - - 55
Medical Progress—Pulmonary Tuberculosis (*continued*) - - 56
Progress in Surgery—Abdominal Surgery - - - - - 58
New Drugs and Praparations - - - - - - - 59
New Appliances and Things Medical - - - - - - 60

**The Institutional Workshop—**
Middlesex Hospital Festival Dinner - - - - - - 61
Practical Books for Hospital Workers - - - - - 63
Practical Departments - - - - - - - - - 63
Construction Notes - - - - - - - - - - 64

EXTRA SUPPLEMENT.—THE NURSING MIRROR.

**News from the Nursing World.**—"The Hospital" Convalescent Fund—A Musical Treat—Uncertain Quantities—Useful Lessons—A Handsome Gift—Comfortable Quarters—Small Objections—Liberal Helpers—Neglected at Night—Queen's Nurses in Scotland—A Meeting at Droylsden—The Alice Fisher Alumnæ—Heroic Deeds—Helping Other Nurses—A Convalescent House—Short Items - - - - - - - - - xxi

**On General Nursing.** VIII.—Pneumonia - - - - - xxiii

**The Friends and Foes of Nurses** - - - - - - xxiv

**The Indian Nursing Service** - - - - - - - xxiv

**The History of Nursing** - - - - - - - - xxiv

**Women Volunteers** - - - - - - - - - - - xxv
**The Mutual Relations of Nurses and Medical Women.** By Mrs. Scharlieb, M.D. II.—Teacher and Pupil - - xxvi
**Nursing in Philadelphia—Notes from Melbourne** - - xxvii
**Notes from America** - - - - - - - - - xxix
**A Spanish Hospital—Presentations** - - - - - xxviii
**The Muses' Looking-Glass**—Where to Spend a Holiday—Ladercost Priory - - - - - - - - - - xxix
**Where to Go—Notes and Queries** - - - - - - xxix
**Everybody's Opinion** - - - - - - - - - xxx
**For Reading to the Sick** - - - - - - - - xxx

